# Comparative Study of the *Marinobacter hydrocarbonoclasticus* Biofilm Formation on Antioxidants Containing Siloxane Composite Coatings

**DOI:** 10.3390/ma15134530

**Published:** 2022-06-27

**Authors:** Todorka G. Vladkova, Deyan M. Monov, Danail T. Akuzov, Iliana A. Ivanova, Dilyana Gospodinova

**Affiliations:** 1 Department of Polymer Engineering, Faculty of Chemical Technology, University of Chemical Technology and Metallurgy, 1756 Sofia, Bulgaria; akuzov@gmail.com; 2 Faculty of Biology, Sofia University Saint Kliment Ohridski, 1164 Sofia, Bulgaria; deyanmoni@gmail.com (D.M.M.); ilivanova@abv.bg (I.A.I.); 3 Electrical Faculty, Technical University of Sofia, 1000 Sofia, Bulgaria; dilianang@tu-sofia.bg

**Keywords:** *Marinobacter hydrocarbonoclasticus* biofilm, low adhesive siloxane coatings, effects of six antioxidants

## Abstract

No systematic study of antioxidant containing coatings and their anti-biofilm action has been reported so far. The utilization of antioxidants in protective coatings to inhibit marine biofilm formation is a current challenge. The aim of this preliminary study was to prepare, characterize and compare the efficiency of low adhesive siloxane composite coatings equally loaded with different antioxidants against mono-species biofilms formation. Most often participating in the marine biofilms formation, *Marinobacter hydrocarbonoclasticus* was the test bacterium. Both the biofilm covered surface area (BCSA) and corrected total cell fluorescence (CTCF) (by fluorescent microscopy) were selected as the parameters for quantification of the biofilm after 1 h and 4 h incubation. Differing extents of altered surface characteristics (physical-chemical; physical-mechanical) and the specific affection of *M. hydrocarbonoclasticus* biofilm formation in both reduction and stimulation, were found in the studied antioxidant containing coatings, depending on the chemical nature of the used antioxidant. It was concluded that not all antioxidants reduce mono-species biofilm formation; antioxidant chemical reactivity stipulates the formation of an altered vulcanization network of the siloxane composites and thus microbial adhesion which influences the surface characteristics of the vulcanized coatings; and low surface energy combined with a low indentation elastic modulus are probably pre-requisites of low microbial adhesion.

## 1. Introduction

Microbial adhesion followed by biofilm formation is a common, non-desirable phenomenon of any living or nonliving material surface in contact with microbial species. Biofilm formation is the initial step of the complex marine biofouling process limiting the performance of submerged surfaces in numerous applications.

A variety of approaches (physical, physical-chemical and enzymatic) to reduce marine biofilm formation are currently known, including many approaches that are biomimetic and/or based on the use of natural derivatives, such as natural biocides, surfactants, quorum-sensing inhibitors and others [1,2,3]. Unfortunately, no report could be found in the literature about surfaces that are able to completely stop the development of marine biofilm, even if they contain biocide.

The deposition of relevant coatings is one of the most often-used approaches in the creation of materials that reduce biofilm formation. Low adhesive, fouling release, siloxane composite coatings are currently the most promising non-toxic alternative to the biocide-containing anti-biofouling paints, which are already banned because of their toxicity. Milne [4] was among the first researchers who pointed out the antifouling properties of siloxane (silicone) polymers. This observation constitutes the basis of most siloxane fouling release coatings that facilitate only the weak adhesion of macro-fouling organisms and ensure the self-cleaning of high speed moving ships (15 knot and above) by easy detachment (release). Siloxane composite coatings, preventing macro fouling of any submerged surfaces, including statically immersed ones, were successfully developed later [5]. However, all known siloxane coatings only partially inhibit biofilm formation and full inhibition remains a significant challenge.

The idea to improve the anti-biofilm activity of low adhesive siloxane composite coatings by including antioxidants in their composition arose from knowledge about the non-reversible macrofoulers’ (mussels and others) attachment by oxidative cross-linking of adhesive proteins secreted by them [6,7,8]. The microbial exopolymeric substances (EPSs) differ significantly from those secreted by macrofoulers, but if biofilm formation is a result of oxidative EPSs cross-linking it could be inhibited by relevant antioxidants. There are no convincing data in the literature indicating oxidative reactions participate in biofilm formation, but the improved anti-settlement properties in the presence of antioxidants, signals that oxidative processes are fundamental for bioadhesion. For example, it was experimentally demonstrated that *Streptococcus mutans* biofilm formation [9] was reduced by the following antioxidants: Gallic acid, Ascorbic acid, Quercetin, Tannic acid and Salicylic acid supposedly be due to the inhibition of the following exopolymer-producing enzymes: Glycosylic transferase and Fructose transferase, i.e., the reduction of biofilm formation is possible via the inhibition of exopolymers-producing enzymes by means of antioxidants.

Supposing that the cross-linking mechanism of the microbial EPSs could be oxidative, a non-toxic antioxidant was included in low adhesive, antifouling siloxane coatings and was expected to further reduce biofilm formation on their surface. In 2013, the increased anti-multispecies biofilm action in a Mediterranean aquarium was first reported [10], and confirmed in Black Sea equatorial [11], which was viewed as an indication for the oxidative crosslinking of microbial EPSs [10,11].

Discussions of antioxidant coatings as a new, environmentally friendly alternative of the marine biocide containing antifouling paints have already been published [12,13].

Different chemical classes of natural and synthetic compounds including amino acids, peptides, terpenoids, polyphenols, vitamins [14], graphene materials [15], etc., are known that demonstrate antioxidant activity. They are widely studied for application in the cosmetic, pharmaceutical and food industry among others. Hutch research reports on the biosynthesis evolution of emergent marine antioxidants, their functional and ecological role in the ocean, their biotechnological production and their potential applications as new drugs, dietary supplements and health care products [14].

The literature presents scarce systematic studies on the anti-biofilm action of antioxidants in marine protective coatings. The utilization of antioxidants to reduce biofilm formation on marine coatings remains a current challenge. This motivated us to perform a comparative study on the ability of different antioxidants to reduce marine biofilm formation on low adhesive siloxane antifouling coatings starting with mono specie biofilm of the Gram-negative bacterium, *Marinobacter hydrocarbonoclasticus* (*M. hydrocarbonoclasticus*), which is one of the species most often participating in marine biofilms formation and extensively used as a model in marine bio-fouling research [16].

Thus, the aim of this preliminary investigation was the preparation, characterization and evaluation of *M. hydrocarbonoclasticus* biofilm formation on low adhesive siloxane composite coatings, containing the same amount of different antioxidants, to compare their anti-biofilm efficiency. Fluorescent microscopy was selected as the tool for the anti-biofilm efficiency quantitation with the following two parameters: biofilm coated surface area (BCSA) and corrected total cell fluorescence (CTCF), corresponding to the attached bacterial cells.

## 2. Materials and Methods

The effects of both the type of the antioxidant and time of exposure (1 h and 4 h) on the initial attachment of *M. hydrocarbonoclasticus* cells were evaluated using different chemical nature oil-soluble antioxidants included in a basic siloxane low adhesive composite coating [5] at the same loading level of 2 wt.%. For comparison, the biofilm formation on bare glass and a glass sample covered with the same siloxane coating without the antioxidant were studied.

### 2.1. Coating Compositions

The coating compositions used in this investigation are based on room temperature vulcanizing (RTV) siloxane elastomers (Gelest, Morrisville, PA, USA); crosslinking agent (ES40, PSI-021, Gelest, Morrisville, PA, USA); catalyst dibutyltin-dilaurate (SND 3260, Gelest, Morrisville, PA, USA); and 2 wt.% antioxidant:

- Butylated hydroxyanisole (Sigma Aldrich, St. Louis, MO, USA);
- α-Tocopherol (E307, Panteley Toshev Ltd., Sofia, Bulgaria);
- Ethyl cinnamate (Sigma Aldrich, St. Louis, MO, USA);
- L-Ascorbil palmitate (oil soluble vitamin C; Sigma Aldrich, St. Louis, MO, USA);
- DL-Tioctic acide (Lipoic acid; ZeinPharm, Nauheim, Germany);
- Dodecyl gallate (E312, Sigma Aldrich, St. Louis, MO, USA).

All coating compositions were prepared as described in [5].

### 2.2. Coated Test-Samples

Glass plates (10 × 10 × 2 mm) were spin-coated (at 400 min^−1^) with a primer consisting of ethyl-triacetoxysilane (50 wt.% toluene solution) and a catalyst (3 wt.% dibutyltin-dilaurate) to provide good adhesion of the coating to the glass surface. The primed dry glass plates were then spin covered with a corresponding composition under the same conditions. Prior to testing, the prepared test-samples were kept under ambient room conditions for 30 days to be cross-linked. The thickness of the dry coating was 220–240 µm as measured by a stereomicroscope Leica MZ16 FA (Leica, Wetzlar, Germany).

The test samples were numbered as follows:

(1) Bare glass sample;
(2) Control—glass sample, coated with siloxane composition without antioxidant;
(3) Glass sample, coated with siloxane composition, containing 2 wt.% Thioctic acid;
(4) Glass sample, coated with siloxane composition, containing 2 wt.% Butylated hydroxianysole;
(5) Glass sample, coated with siloxane composition, containing 2 wt.% α-Tocopherol;
(6) Glass sample, coated with siloxane composition, containing 2 wt.% Ethyl cinnamate;
(7) Glass sample, coated with siloxane composition, containing 2 wt.% L-Ascorbil palmitate;
(8) Glass sample coated with siloxane composition, containing 2 wt.% Dodecyl gallate.

### 2.3. Water Contact Angle (WCA) and Surface Energy (γ)

The contact angle-measuring instrument Easy Drop (Kruss, Hamburg, Germany) was employed for static contact angle measurements (angle resolution ± 0.10) using the following three liquids with known surface tension: water, ethylene glycol, and n-hexadecane. The surface energy (Ec) was calculated according to Fowkes’ method [17].

### 2.4. Atomic Force Microscopy (AFM)

Easyscan 2 apparatus equipped with a Pointprobe Contr-10 silicone SPM sensor (Nanosurf, Liestal, Switzerland; dimensions of 2 × 450 × 50 µm^3^) was employed to obtain plane and 3D images of the investigated dry surfaces operating in the contact mode. Diamond Vicker’s pyramid (Nanosurf, Liestal, Switzerland) with a pike angle of 136° was used for all measurements at room temperature, with a loading speed of 0.250 mN/s.

### 2.5. Depth Sensing Indentation (DSI)

A dynamic Ultra Micro-Hardness Meter DUH-211 S (Shimatzu, Kyoto, Japan) was employed to evaluate the indentation hardness (HIT), Vicker’s hardness (VIH), and indentation elastic modulus (EIT) under the following conditions: test force of 0.45 mN; loading speed of 6.0 (0.0250) mN/s; penetration depth of 25 nm.

### 2.6. Test Bacterium and Bacterial Biofilm Formation

*Marinobacter hydrocarbonoclasticus* DSM 50418 (*M. hydrocarbonoclasticus*; Gram-negative, aerobic, rod-shaped marine bacterium; size of about ±2 μm; growth in the temperature range of 10 to 42 °C; other name *Cobetia marina*) was the test bacterium for this study, provided by the National Bank of Microorganisms and Cell Cultures (NBMCC), Sofia, Bulgaria. The test procedure included the sterilization of all samples with isopropanol 70% for 30 min under ultraviolet light; 1 h incubation with 4.0 × 10^7^ CFU/mL *M. hydrocarbonoclasticus* in 5 mL suspension on an orbital shaker (50 rpm) and then washing in artificial seawater (ASW; Tropic Marine^®^, pH 7, 33.3 g/L ultrapure water, Dr. Biener GmbH, Wartenberg, Germany). The *M. hydrocarbonoclasticus* culture was diluted in a minimal medium (1:100 marine broth to ASW) in order to obtain an optical density (O.D.) of 0.1 at a wavelength of 600 nm, which corresponds to 4.0 × 10^7^ cfu/mL. Bare sterile glass and coated sterile glass samples exposed to a suspension of *M. hydrocarbonoclasticus* were the negative control, while bare sterile glass and the corresponding coated sterile samples exposed to a suspension without *M. hydrocarbonoclasticus* were the positive control. After the exposure time (1 h and 4 h), the bacterial suspensions were removed and all samples were quickly immersed in artificial salt water (ASW) to remove the excess of non-adhered cells. The test plates were fixed with 5 mL glutaraldehyde (2.5% in ASW) for 20 min at room temperature; afterwards, they were washed once again with ASW for 1 min on a plate shaker. After 24 h drying at room temperature, the plates were ready for staining. All experiments were duplicated for the coatings with the same composition.

### 2.7. Fluorescence Microscopy

Fluorescence microscopy was used for the quantitative evaluation of the biofilm formation on composite siloxane coatings containing the same amount (2 wt.%) of the following antioxidants of different chemical natures: Thioctic acid; Butylated hydroxyanisole; α-Tocopherol; Ethyl cinnamate; L-Ascorbic acid 6-palmitate; and Dodecyl gallate.

The coated test samples were stained with diamino-2-phenyl-indol (DAPI, Molecular Probes, Invitrogen, CA, USA) and kept for 10–15 min in the dark for coloration. After triple washing with phosphate buffer (PBS), they were observed using a Fluorescence microscope (Leica DM 5500B, Leica Microsystems manufacturer, Vienna, Austria) equipped with an integrated camera. Around 25–30 images were captured for every coated sample, evenly distributed on the sample surface and processed by Fiji software, Image J 1.53q, W. Rasband et al, National Institutes of Health, USA [18] measuring the biofilm coated surface area (BCSA) and its fluorescence (corrected by the fluorescence of the control sample with coatings without antioxidants). Based on these data, the corrected total cell fluorescence (CTCF) was calculated as a quantitative measure for the adhered bacterial cells.

## 3. Experimental Results and Discussion

**Figure 1:**
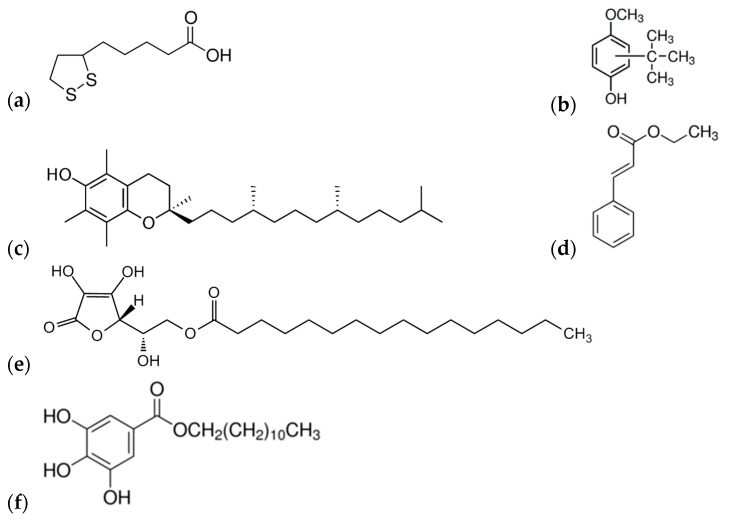
Structural formulas of the used antioxidants: (**a**) Thioctic acid (α-Lipoic acid; natural product; hydrogen-transferring co-factor); (**b**) Butylated hydroxyanisole (synthetic antioxidant); (**c**) α-Tocopherol (Vitamin E; E307 in food); (**d**) Ethyl cinnamate (Ethyl (2Z)-3-phenylprop-2-enoate); (**e**) L-Ascorbic acid 6-palmitate (oil soluble vitamin C); and (**f**) Dodecyl gallate (food additive E312; antioxidant and preservative).

Different classes of chemical compounds demonstrate antioxidant activity but their anti-biofilm activity is rarely studied. With the expectation that the chemical nature could have a significant influence on the inhibition of *Marinobacter hydrocarbonoclasticus* biofilm formation, six types of liquid antioxidants were used in this study for which the structural formulas are presented in Figure 1. *M. hydrocarbonoclasticus* biofilm formation was investigated using siloxane composite coatings containing the same amount (2 wt.%) of one of the antioxidants presented in Figure 1.

### 3.1. Surface Characteristics of the Coated Test Samples

Knowing that microbial colonization on solid surfaces can be affected by surface physical-chemical (hydrophilic/hydrophobic balance, surface tension, roughness) [19,20,21,22,23] and physical-mechanical parameters [24,25,26] as well as expecting that the including of antioxidants in the coating compositions could affect these parameters, the surface characterization of each test sample was carried out before testing the biofilm formation. The results are presented in Table 1

A comparison of both the physical-chemical (WCA, Ec, Ed, Ep, Ra, Rq) and physical-mechanical (HMV, HIT and EIT) surface characteristics of the samples 3 to 8 (containing 2 wt.% different antioxidants) to those of the control sample 2 (without antioxidant) demonstrates that the surface parameters were affected to different extents dependent on the chemical nature of the antioxidant.

It is evident that the Ethyl cinnamate (Table 1, Sample 6) in the coating composition leads to the highest WCA increase (up to 107.2 ± 0.6°), compared to that of the control without antioxidants (Table 1, Sample 2) of 104.1 ± 0.3°. All other antioxidants decrease the WCA of the corresponding coating, most significantly (down to 91.1 ± 0.7°) for that containing L-ascorbic palmitate (Table 1, Sample 7). All coated surfaces with a WCA of higher than 90° are hydrophobic (Table 1, row 1, Samples 2–8) and their surface energy, Ec, excluding Sample 7 (Table 1, Samples 2–6, 8) is in the range of the so called “Bayer’s window” (Ec of 20 mN/m–25 mN/m) accepted as optimal for a good biofouling release [27].

Although some slight deviations were observed, dependent on the presence of different antioxidants in the coatings, the surface roughness, Ra and Rq of the coated samples (Table 1, rows 5 and 6, Samples 2–8) remained in the nanoscale range, whereas the *M. hydrocarbonoclasticus* cells were rood shaped with sizes in the micron scale. This makes their entry into “nano-valleys” impossible. Secreted EPSs could only penetrate in such nano-ruffle surfaces to influence the initial attachment of the *M. hydrocarbonoclasticus* cells.

The physical-mechanical parameters were as follows: dynamic Vicker’s hardness, HMV, indentation hardness, HIT and indentation elastic modulus, EIT (Table 1, the last 3 rows, Samples 2–8) which were also influenced by the presence of different antioxidants in their composition. This indicates differences in their vulcanization networks, most probably due to the participation of the antioxidant in the cross-linking of the siloxane composite. Knowing that the low elastic modulus contributes to lower bio adhesion [26], it could be expected that the lower EIT values contribute to a decreased *M. hydrocarbonoclasticus* adhesion. The EIT was below that of the control sample without antioxidants (Table 1, Sample 2) and the lowest in the presence of Ethyl cinnamate (Table 1, the last row, Sample 6).

### 3.2. Biofilm Formation by Marinobacter hydrocarbonoclasticus

The spread of *M. hydrocarbonoclasticus* (*Cobetia marina*) in marine ports’ bacterium usually forms structured biofilms on hydrophobic surfaces [16]. The biofilms are usually inhomogeneous in the spreading and thickness, and are both changeable with the time of the cell growth (see Supplementary Figure S1). Therefore, in this comparative study, both the biofilm coated surface area (BCSA) and corrected total cell fluorescence (CTCF) were used to evaluate the effect of different classes of chemical compounds with antioxidant activity on *M. hydrocarbonoclasticus* biofilm formation after 1 h and 4 h incubation. The testing was performed on spin covered glass samples with siloxane coating containing the same amount (2 wt.%) of different antioxidants (Samples 3–8) or without antioxidants (Sample 2) and bare glass (Sample1), with the last two used for comparison.

**Figure 2:**
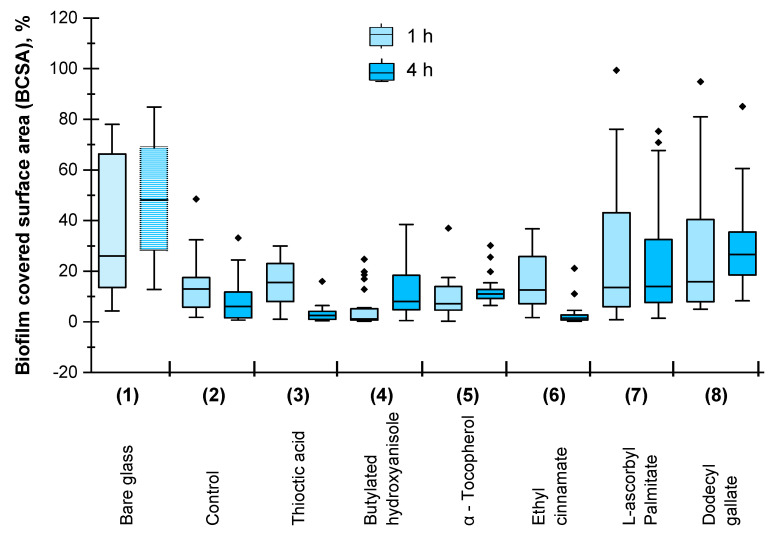
Biofilm covered surface area (BCSA), % on bare glass (Sample 1); control, glass with siloxane composite coating without antioxidants (Sample 2) or containing different antioxidants: Thioctic acid (Sample 3); Butylated hydroxyanisole (Sample 4); α-Tocopherol (Sample 5); Ehthyle cinnamate (Sample 6); L-ascorbile palmitate (Sample 7); Dodecyl gallate (Sample 8).

**Figure 3:**
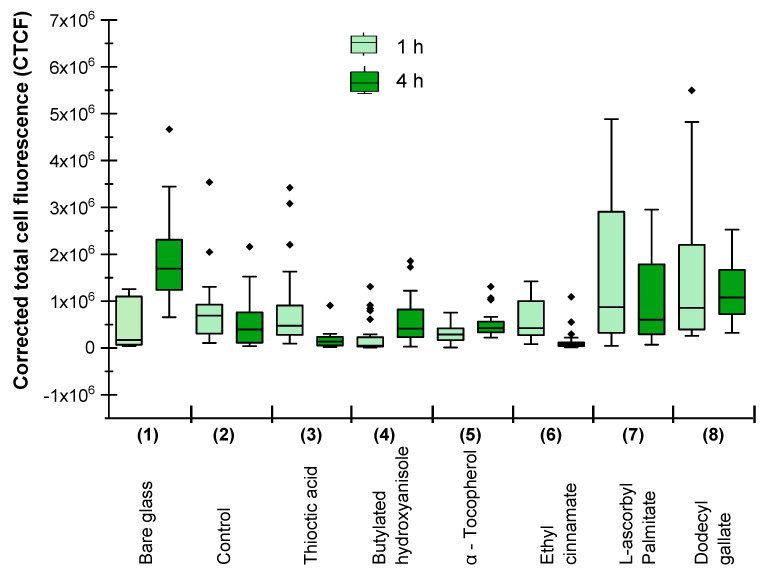
Corrected total cell fluorescence (CTCF) on bare glass (Sample 1); control, glass with siloxane composite coating without antioxidants (Sample 2) or containing different antioxidants: Thioctic acid (Sample 3); Butylated hydroxyanisole (Sample 4); α-Tocopherol (Sample 5); Ehthyle cinnamate (Sample 6); L-ascorbile palmitate (Sample 7); Dodecyl gallate (Sample 8).

Figure 2 and Figure 3 demostrate the specific *M. hydrocarbonoclasticus* antibiofilm effect (evaluated by BCSA and CTCF, respectively) of antioxidants with a variety of chemical structures selected for this investigation. Some trends in the effects of the different antioxidants were clearly observed, although with significant deviations (maybe due to more or less homogenues dispersion of the antioxidant in the polymer matrix) in the observed BCSA (Figure 2) and the CTCF (Figure 3) after 1 h (the light blue and light green bars, respectively) and 4 h of incubation (the dark blue and dark green bars, repectively).

Confluent biofilm does not form on any coated surface or bare glass (BSCA is below 100% in all cases) and the effect of the different antioxidants on *M. hydrocarbonoclasticus* biofilm development is specific, as is evident in Figure 2. Compared to the bare glass (Figure 2, Sample 1), all coated surfaces (Figure 2, Samples 2–8) reduced BCSA after 1 h (the light blue bars) and 4 h growth (the dark blue bars) indicating some anti-biofilm activity of all coatings, including that without antioxidants (Figure 2, Sample 2). This is not a surprise as the basic coating composition forms low adhesive fouling release coatings, and the addition of antioxidant aims at improving the performance of this type of coating. The effect of antioxidant-containing coatings (Figure 2, Samples 3–8) on the BCSA is quite different, as compared to the control coating without antioxidants (Figure 2, Sample 2):

- The average BCSA (the black line in the bares) is lower for the samples for which coatings contain Thioctic acid, Butylated hydroxyanisol or Ethyl cinnamate (Figure 2, Sample 3, Sample 4, Sample 6, respectively) indicating their inhibiting effect on biofilm formation after 1 h and 4 h growth;
- The average BCSA (the black line in the bares) is higher for the samples for which coatings contain L-Ascorbic palmitate and Dodecyl gallate (Figure 2, Sample 7, Sample 8, respectively), thereby indicating that these antioxidants stimulate 1 h and 4 h biofilm development.
- The average BCSA (Figure 2, the black line in the bares) decreases after 4 h (the dark blue bars) as compared to 1 h of *M. hydrocarbonoclasticus* cells growth (the light blue bars) for the control coating without antioxidant (Figure 2, Sample 2) and of those containing Thioctic acid or Ethyl cinnamate (Figure 2, Samples 3, Sample 6); however, it increases on bare glass (Figure 2, Sample 1) and on the coatings containing butilated hydroxianisol, α-Tocopherol, L-Ascorbil palmitate and Dodecyl gallate (Figure 2, Sample 4, Sample 5, Sample 7 and Sample 8, respectively).
- Promising results regarding *M. hydrocarbonoclasticus* biofilm development suppression were found for Thioctic acid and the Ethyl cinnamate. The average BCSA of coatings containing these antioxidants (Figure 2, Samples 3 and Sample 6) is lower than that of the control coating (Figure 2, Sample 2) after 1 h (light blue bars) and decreases after 4 h *M. hydrocarbonoclasticus* growth (dark blue bars).

The CTCF data, presented in Figure 3, confirm the specific effect of the different antioxidants found by BCSA:

- The average CTCF (the black line in the bars) on bare glass (Figure 3, Sample 1) increases after 4 h (the dark green bar) compared to 1 h of *M. hydrocarbonoclasticus* growth (the light green bar), as it did for BCSA (Figure 2, Sample 1)
- The average CTCF (the black line in the bars) of the samples containing Thioctic acid, Butylated hydroxyanisol, α-Tocopherol or Ethyl cinnamate (Figure 3, Samples 3–6) is below than that of the control without antioxidants (Figure 3, Sample 2) after 1 h (Figure 3, the light green bars) as well as after 4 h (Figure 3, the dark green bars) of *M. hydrocarbonoclasticus* incubation; for the coated samples containing L-Ascorbic palmitate or Dodecyl gallate (Figure 3, Samples 7 and Sample 8)) it was higher.
- The average CTCF (the black line in the bars) decreased after 4 h (the dark green bars) compared to 1 h (the light green bars) of *M. hydrocarbonoclasticus* incubation for Samples 2, 3, 6 (Figure 3). This indicates an expected releasing effect of the control siloxane coating without antioxidants (Figure 3, Sample 2) and an improvement of this effect by the presence of both Thioctic acid or Ethyl cinnamate (Figure 3, Sample 3 and Sample 6, respectively), demonstrating a bactericidal activity.
- The average CTCF (the black line in the bars) is lower than that of the control sample (Figure 3, Sample 2) for the coatings containing Thioctic acid or Ethyl cinnamate (Figure 3, Sample 3, Sample 6) and decreases after 4 h (the dark green bars) compared to 1 h of *M. hydrocarbonoclasticus* incubation (the light bars).

As found in a former study [10,11], the α-Tocopherol in siloxane composite coatings insignificantly reduces *M. hydrocarbonoclasticus* biofilm formation, although the reduction in multi-species biofilm formation in Mediterranean aquarium and Black Sea was significant. In agreement with the results of the former investigation [11], the effect of the α-Tocopherol was found to be insignificant for BCSA (Figure 2, Sample 5 compared to the control) and CTCF (Figure 3, Sample 5 compared to the Control) in this investigation.

The most active sample regarding the inhibition of *M. hydrocarbonoclasticus* biofilm formation on the studied low adhesive siloxane composite coatings was Sample 6, containing 2 wt.% Ethyl cinnamate followed by Sample 3 containing 2 wt.% Thioctic acid. The average BCSA (Figure 2) was of 11.8% and 12.3%, respectively, at the first hour (the light blue bars); 4.2% and 4.6%, respectively, at the fourth hour (the dark blue bars); the average CTCF (Figure 3) was 3.0 × 10^6^ and 3.6 × 10^6^, respectively, at the firs hour (the light gree bars) and 1.3 × 10^6^ and 2.6 × 10^6^, respectively, at the fourth hour (the dark green bars).

Both, the average BCSA and CTCF of Sample 6 and Sample 3 were less than those of the control (without antioxidant) and all other samples whereas the average BCSA and CTCF of Sample 8 (containing Dodecyle gallate) and Sample 7 (containing L-Ascorbyl palmitate) were higher compared to those of all other covered samples.

The most active in the reduction of *M. hydrocarbonoclasticus* biofilm formation was the sample containing Ethyl cynnamate (Table 1, Sample 6) characterized with a higher WCA (of 107.2° ± 0.6), lower surface energy, Ec (of 19.3 mN/m) and lower indentation elastic modulus (of 0.99 N/mm^2^) as compared to the corresponding parameter of the control sample without antioxidants (WCA of 104.1°; Ec of 21.4 mN/m; and EIT of 1.76 ± 0.06 N/mm^2^) (Table 1, Sample 2). Sample 7 (containing L-Ascorbic palmitate) and Sample 8 (containing Dodecyle gallate), which stimulated *M. hydrocarbonoclasticus* biofilm formation, were characterized with lower WCA (91.1° and 99.3°, respectively), higher surface energy, Ec (25.9 mN/m and 24.0 mN/m, respectively) and higher indentation elastic modulus, EIT (5.61 N/mm^2^ and 3.93 N/mm^2^, respectively) compared to the control sample without antioxidant (Table 1, Sample 2). It seems that the combination of low surface energy, Ec and low indentation elastic modulus, EIT is a pre-requisite of low microbial adhesion, as it is for the bioadhesion of macro biofoulers [21,22,25,26].

The observed differences in the effect of the different antioxidants on the antibiofilm activity of the studied low adhesive siloxane coatings could be connected to different alterations of influencing the bioadhesion and biofilm formation surface physical-mechanical (maily indentation elastic modulus) and physical-chemical parameters (surface energy and related parameters), which are presented in Table 1. This effect could be due to a possible participation of the antioxidants in the hydrosilation cross-linking of the siloxane composites and the formation of specific vulcanization networks due to their different chemical reactivity. The last parameters are indicated by the changes in the Vikers’ dynamic surface hardness (HMV), indentation hardness (HIT) and indentation elastic modulus (EIT), surface roughness, Ra and Rq, as well as surface energy, Ec and related parametes. The mechanism of the action clearance of every antioxidant requires further in-depth study.

## 4. Conclusions

The same amount of different antioxidants alters bioadhesion, thereby influencing the surface characteristics (physical-chemical and physical-mechanical) of the studied low-adhesive siloxane composite coating to different extents. This effect is stipulated most probably by the specific vulkanization network formation due to the different chemical reactivity of the tested antioxidants. The altered surface characteristics alter the anti-biofilm activity of the coated samples.

Not all antioxidants assist in the anti-biofilm activity of the coated surfaces. Their effect is specific, as some of them, such as Etyle cinnamate and DL-Thiotic acid, significantly inhibit its formation whereas others, such as L-ascorbic acid and Dodecyl gallate, stimulate *M. hydrocarbonoclasticus* biofilm formation on low adhesive siloxane coatings.

The most effective treatment against *M. hydrocarbonoclasticus* biofilm formation, among the six tested coatings, appeared to be the one containing 2 wt.% Ehylcinnamate, indicated by the lowest biofilm covered surface area (BCSA) and lowest corrected total cell fluorescence (CTCF) after 1 h and 4 h of *M. hydrocarbonoclasticus* incubation.

The combination of low surface energy, Ec and low indentaion elastic modulus, EIT, is probably the pre-requisite for low microbial adhesion.

## Figures and Tables

**Table 1:** Surface physical-chemical characteristics: water contact angle (WCA), surface energy (E_c_), disperse (E_d_) and polar (E_p_) components; surface roughness (Ra, Rq) and physical-mechanical parameters: dynamic Vicker’s hardness (HMV), indentation hardness (HIT), and indentation elastic modulus (EIT) of the studied coatings: (2)—Control without antioxidant; or containing 2 wt.%: (3)—DL-Tioctic acid; (4)—Butylated hydroxyanisol; (5)—α-Tocopherol; (6)—ethyl cinnamate; (7)—L-ascorbil palmitate; (8)—dodecyl gallate.

Parameter	Coated Glass Sample No.						
	2 Control	3 DL-Thiotic Acid	4 Butylated Hydroxyl Anisole	5 α-Tocopherol	6 Ethyl Cinnamate	7 L-Ascorbic Palmitate	8 Dodecyl Gallate
WCA, °	104.1 ± 0.3	99.9 ± 0.2	101.1 ± 0.7	92.5 ± 0.4	107.2 ± 0.6	91.1 ± 0.7	99.3 ± 0.1
Ec, mN/m	21.4	24.8	22.0	24.6	19.3	25.9	24.0
Ed, mN/m	19.8	22.9	20.8	22.0	18.2	23.2	23.2
Ep, mN/m	1.6	1.9	1.2	2.3	1.1	2.7	0.8
Ra, nm	12 ± 4	72 ± 14	29 ± 5	14 ± 6	14 ± 3	48 ± 11	59 ± 12
Rq, nm	15 ± 7	102 ± 18	61 ± 12	12 ± 9	11 ± 6	65 ± 16	83 ± 17
HMV, N/mm^2^	0.13 ± 0.02	0.46 ± 0.09	0.20 ± 0.09	0.17 ± 0.06	0.15 ± 0.03	0.33 ± 0.02	0.28 ± 0.01
HIT, N/mm^2^	0.34 ± 0.01	0.51 ± 0.01	0.42 ± 0.06	0.22 ± 0.07	0.29 ± 0.01	0.67 ± 0.03	0.42 ± 0.05
EIT, N/mm^2^	1.76 ± 0.06	1.32 ± 0.04	2.05 ± 0.10	2.39 ± 0.09	0.99 ± 0.12	5.61 ± 0.02	3.93 ± 0.06

## Data Availability

Not applicable.

## Supplementary Materials

The following supporting information can be downloaded at: https://www.mdpi.com/article/10.3390/ma15134530/s1, Figure S1 Fluorescent microscopy picture of low adhesive siloxane coating containing 2 wt. % butylated hydroxyanisole: (a)–after 1 h exposure and (b)–after 4 h exposure to the Marinobacter hydrocarbonoclasticus suspension.
