# Supplementary material for: Comparative Study of the Marinobacter hydrocarbonoclasticus Biofilm Formation on Antioxidants Containing Siloxane Composite Coatings
Source: Materials (Basel). 2022 Jun 27;15(13):4530. doi: 10.3390/ma15134530 (PMC9267624; doi:10.3390/ma15134530)
Supplement: Supplementary file 1 [file materials-15-04530-s001.zip › materials-1777791-supplementary.pdf]

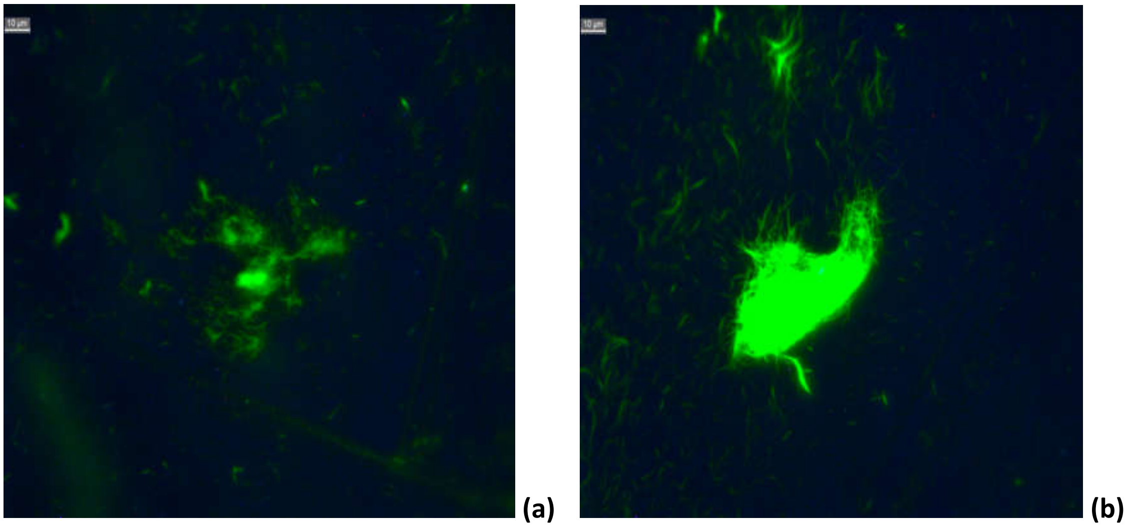

Figure S1. Fluorescent microscopy picture of low adhesive siloxane coating containing 2 wt. % butylated hydroxyanisole: (a) – after 1 h exposure and (b) – after 4 h exposure to the *Marinobacter hydrocarbonoclasticus* suspension.
